# A systematic review of the effects of advance care planning facilitators training programs

**DOI:** 10.1186/s12913-019-4192-0

**Published:** 2019-06-07

**Authors:** Carmen Wing Han Chan, Nancy Hiu Yim Ng, Helen Y. L. Chan, Martin M. H. Wong, K. M. Chow

**Affiliations:** The Nethersole School of Nursing, Faculty of Medicine, The Chinese University of Hong Kong, 6/F, Esther Lee Building, Shatin, NT Hong Kong

**Keywords:** Advance care planning, Palliative care, End-of-life care, Education, Training, Facilitator, Nurse, Healthcare professionals

## Abstract

**Background:**

Advance care planning (ACP) is the process of ongoing communication among patients, family and health care professionals regarding what plans for future care are preferred in the event that patients become unable to make their own decisions. Clinicians play an important role in ACP as both initiators and decision coaches. However, lack of training for clinicians has frequently been reported as the reason for low involvement in ACP discussions - hence the present review evaluates the effectiveness of ACP training programs for healthcare professionals to guide the development of novel training programs for them in the future.

**Methods:**

A literature search for intervention studies was conducted independently by two reviewers in July 2018. Participants included all healthcare professionals working with adult patients suffering from terminal illness. The primary outcomes were the professionals’ knowledge of and attitudes towards ACP, and self-perceived competence in ACP conversations. The Effective Public Health Practice Project appraisal tool was used to examine the quality of the studies included.

**Results:**

A total of 4025 articles were identified, and ten eligible articles, covering 1081 participants, were included in the review. However, there is a lack of high quality randomized controlled trials of providing ACP training for nurses working in non-palliative care hospital settings. The overall quality of the intervention studies was moderate. All the studies included used instructional sessions in their interventions, while some contained group discussion, role-play and the use of advanced technology. The training programs increased the knowledge, attitudes towards shared decision-making, perceived communication skills, confidence, comfort and experiences concerned with discussing end-of-life (EOL) issues. Patient advocacy, job satisfaction and perceived level of adequate training for EOL care were improved. The use of ‘decision aids’ was rated as acceptable and clinically useful.

**Conclusions:**

Training for healthcare professionals in ACP has positive effects on their knowledge, attitude and skills. The use of decision aids and advanced technology, instructional sessions with role play, training content focused on ACP communication skills and the needs and experience of patient in the ACP process, and a values-based ACP process are all those factors that made the ACP training programs effective.

## Background

Palliative care focuses on the quality of life of patients and their families facing a life-threatening illness, through the prevention and relief of pain and other problems, physical, psychosocial and spiritual [1].

According to the statistics of one regional hospital in Hong Kong, the rate of deaths in acute, non-palliative settings was 91.7% in 2016, meaning that a significant proportion of deaths occurred in non-palliative care units such as acute medical and surgical units. Healthcare professionals, especially doctors and nurses, working in non-palliative care units and acute care settings would be expected to provide EOL care. Communication and decision-making about the goals of care as identified by seriously ill hospitalized patients and their families are important elements for improvement in the quality of end-of-life (EOL) care [2].

Advance care planning (ACP) is the process of ongoing communication among patient, family and healthcare professional regarding the preferred planning for future care in the event when patients become unable to make their own decisions. Healthcare professionals are expected to play important roles to initiate ACP and act as decision coaches [3]. ACP provided by trained non-physician facilitators have been shown to increase the convergence of patients’ wishes and the EOL care that they receive [3].

An advance directive (AD) is a statement, usually in writing, made by a person to indicate advance refusal of medical treatment and directions on the kind of life-sustaining treatments that should be withheld/withdrawn when he/she is no longer mentally capable of making healthcare decisions. The literature shows that facilitated ACP can increase the number of meaningful and valid ADs, strengthen patient autonomy and improve the quality of care when EOL is near [4–6]. A systematic review by Brinkman-Stoppelenburg, Rietjens and van der Heide [7] found that comprehensive ACP discussion may be more effective than ADs alone in improving compliance with a patient’s EOL wishes and satisfaction with care. Findings of the systematic review by Klingler, in der Schmitten and Marckmann [8] even showed that facilitated ACP has the potential to reduce the net costs of care.

However, an integrative review by Rietze and Stajduhar [9] found that nurses in acute care settings had a low involvement in ACP discussions. Limited education and training in ACP and EOL care conversations are reported as barriers in facilitating ACP conversations [10]. Lack of education and knowledge, lack of time with patients, communication barriers and symptom management are some of the themes drawn from the literature review by McCourt, Power and Glackin [11] on the provision of EOL care in acute hospital settings.

Lund, Richardson and May [12] suggested that interventions mostly likely to increase the adoption of ACP in clinical practice are those that make elements of ACP workable within complex and time-pressured clinical workflows. The systematic review and meta-analysis by Oczkowski, Chung, Hanvey, Mbuagbaw and You [13] suggested that the use of structured communication tools might increase the communication of preferred care. Another systematic review, by Cardona-Morrell et al. [14], concluded that the available decision aids seemed to enhance patients’ and surrogates’ knowledge of the care options. Yet the studies included in these systematic reviews were not targeted at ACP training programs for healthcare professionals [12–14]. Chung, Oxzkowski, Hanvey, Mbugbaw, and You [15] conducted a systematic review and meta-analysis on educational interventions and suggested that when compared with usual teaching, healthcare professionals’ self-efficacy, knowledge and EOL communication scores might be improved by EOL communication training. However, the finding was only applicable to such training in general, only a very few of the included studies focused on ACP facilitator training [15]. A systematic review was thus conducted to evaluate the effectiveness of ACP training programs for healthcare professionals. The findings will be used to guide the development of a novel program to meet the training needs of healthcare professional and facilitate ACP conversations in acute care settings.

## Objectives

The purpose of this systematic review is to obtain evidence for the effectiveness of an ACP training program for healthcare professionals. The following research questions were to be answered:

1. What is the effectiveness of the program in improving healthcare professionals’ knowledge regarding ACP?
2. What is the effectiveness of the program in improving healthcare professionals’ attitude regarding ACP?
3. What is the effectiveness of the program in improving healthcare professionals’ competence regarding ACP?
4. What is the effectiveness of the program in increasing the frequency of initiating ACP conversations by healthcare professionals?

## Methods

### Eligibility criteria

#### Types of studies

Randomized controlled trials (RCTs) and quasi-experimental studies with a control group, including uncontrolled before-and-after studies in which participants acted as their own control were included. Eligible articles were those written in English in any publication year. Articles written in languages other than English were excluded.

#### Types of participants

Participants were various classes of healthcare professionals who were working with adult patients over 18 with terminal illnesses. Population groups in community settings or home-dwelling patients and neonatal/pediatric patients were excluded.

#### Types of intervention

Advance care planning training program for healthcare professionals were included. Training programs targeting patients or family members were excluded.

#### Types of outcome measures

The outcomes were healthcare professionals’ knowledge of and attitude towards ACP, self-perceived competence in ACP discussion and the frequency of initiating ACP conversations.

### Search strategies

A comprehensive search was conducted in July 2018. Initial keywords relevant to the topic were identified by a search of MEDLINE. A more extensive search using the identified keywords was performed using the following eight databases: MEDLINE, CINAHL, PubMed, EMBASE, Cochrane Library, JBI EBP, PsycINFO, Health and Psychosocial Instruments. The keywords were: (Advance Care Planning OR advance medical planning OR advance health care planning OR advance care plan* OR ACP OR Advance Directive* OR decision making OR treatment decision making OR ethical decision making OR living will* OR funeral preparation) AND (palliative care OR palliative therapy OR palliative treatment OR end of life OR hospice OR terminal care OR terminally ill OR death and dying OR life limiting illness* OR life threatening illness*) AND (nurs* OR health person* OR health professional* OR allied health worker* OR facilitator* OR physician* OR social worker* OR surgeon* OR oncologist*) AND (education* OR train* OR workshop OR facilitation) AND (hospital$ OR in patient* setting OR acute care OR critical care OR intensive care OR rehabilitation OR surgical OR oncolog*). A manual search of the reference list of included articles and relevant journals was undertaken to identify relevant articles for screening.

### Study selection

After the removal of duplicates, two reviewers (NHYN and MMHW) independently performed the initial screening on the titles and abstracts according to the eligibility criteria. Full texts were then obtained to determine the eligibility of the whole studies. Discrepancies were solved by group consensus and a final decision by the third reviewer (CWHC).

### Data extraction and quality appraisal

For each of the included studies, two reviewers (NHYN and MMHW) independently extracted the study characteristics: aim, design, population, intervention, measurement tool and results. The methodological quality of all the studies was assessed by means of the Effective Public Health Practice Project (EPHPP) appraisal tool [16], which evaluates an interventional study in eight domains: selection bias, study design, confounders, blinding, data collection methods, withdrawals and dropouts, intervention integrity and analysis. The judgement of the methodological rating was strong, moderate and weak, as evaluated by two reviewers independently (NHYN and MMHW). Whenever a discrepancy occurred, it was solved by group consensus and a final decision by the third reviewer (CWHC).

## Results

### Study selection

**Fig. 1:**
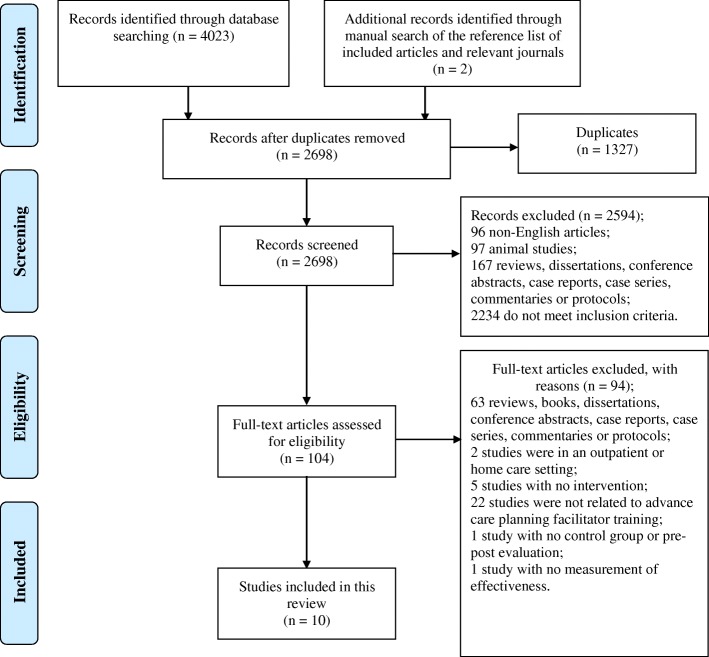
PRISMA flowchart of the study retrieval and selection process

A total of 4025 articles were identified. After the screening on the titles and abstracts, A total of 104 articles were potentially eligible and their full texts were retrieved and reviewed. Finally, ten articles were included in the review [17–26]. A PRISMA flow chart of the study retrieval and selection process with reasons for exclusion at each stage is provided in Fig. 1.

### Participants and settings

The details of the ten studies are given in Table 1. A total of 1081 participants were included in the review. The sample in each study varied from 16 to 278, with five [19, 20, 22, 24, 25] including more than 100 participants. Five studies were conducted in the US [17, 20, 22, 25, 26], two in Australia [19, 24], one in the UK [18], one in Canada [23] and one in Korea [21]. The majority of participants were nurses, physicians and medical students. The participants in three studies were recruited from hospitals [19, 23, 24], in three from internal medicine units [17, 18, 25] including renal medicine [18], in two from critical care units [21, 26] and in another two from students undergoing the usual medical curriculum [20, 22].

**Table 1:** Characteristics of the included studies

First author, year	Aim	Design	Population	Intervention	Measurement tool	Results
Alexander, 2006 [17]	To evaluate the effect of a short course to improve residents’ communication skills in delivering bad news and eliciting patients’ preferences for EOL care	Quasi-experimental study with a non-equivalent control group pretest-posttest design	56 internal medicine residents in the US	1. Intervention group: two-day retreat with 16-h curriculum focusing on (1) symptom management, (2) communication skills and (3) patient experience by small-group discussion, audio-visual materials and role-play 2. Control group: no intervention	1. Audio-recorded encounters with standardised patients before and after the intervention, which was evaluated by Bad-News Conversations and Patient Preferences	1. The intervention group demonstrated statistically significant increases in their overall skill ratings in the delivery of bad news, with improvement in the specific areas of information giving and responding to emotional cues. 2. Although cumulative scores for discussions about patient preferences for treatment did not increase, the intervention group demonstrated enhanced specific skills in EOL decision-making.
Bristowe, 2014 [18]	To develop and pilot a REnal specific Advanced Communication Training programme to advance the communication skills of renal professionals to support people with ESKD to make informed choices about their future care	Pre-test post-test design	16 renal professionals (9 nurses/health-care assistants and 7 consultants) in the UK	One full-day session and two half-day follow-up sessions to address the needs identified in the focus group, with structured sessions around each theme by presentation, discussion and role-play	1. Confidence in communicating about EOL issues with patients (pre-training, immediately post training, at 3 months post training)	1. There was a non-significant increase in confidence in communicating about EOL issues, which was maintained at 3 months.
Detering, 2014 [19]	To develop and evaluate an interactive ACP educational programme for general practitioners and doctors-in-training	Pre-test post-test design	148 doctors from GP and hospital settings (majority the latter) in Australia	The education program on ACP using a DVD, an interactive patient e-simulation, a structured 2 h workshop and a training manual to assist with facilitation of the workshop	Three components assessed before and after the education program: (1) knowledge of ACP; (2) attitudes towards ACP; (3) confidence in discussing ACP	There were no significant differences observed in ACP knowledge following training, and most participants were supportive of patient autonomy and ACP. After training, there was a significant improvement in self-reported confidence in six of eight items.
Greenberg, 1993 [20]	To evaluate the effect of an educational module on third-year medical students	RCT	141 third-year medical student in the US	1. High intervention group: received all the materials given to the low-intervention group, participated in a two-hour seminar during the second week of the clerkship on AD and death and dying experiences, and a follow-up session two weeks later on AD discussion 2. Low intervention group: received two articles pertaining to the DPAHC, a one-page summary of important aspects of the DPAHC a reference bibliography 3. Control group: no intervention, received no material about the DPAHC	1. Knowledge about the DPAHC assessed with 22 true/false/not-sure questions 2. Perceived skill and comfort assessed with 14 questions using a five-point Likert scale ranging from ‘strongly agree’ to ‘strongly disagree’	1. All three groups improved significantly in knowledge about the DPAHC at follow-up. The high-intervention group improved significantly more than the control. or low-intervention group 2. High-intervention group reported more improved perceived skills and comfort and experience with discussing the DPAHC and end-of-life issues.
Jo, 2015 [21]	To examine the effects of an educational program on shared decision-making on EOL care performance, moral sensitivity and attitude towards shared decision-making among Korean nurses	Quasi-experimental study with a non-equivalent control group pretest-posttest design	41 ICU nurses in Korea.	1. Intervention group: educational programme on shared decision-making for 60 min per session, twice a week for 4 weeks 2. Control group: spending the same time but reading the moral guidelines of the hospital	1. End-of-life care performance scale 2. The Moral Sensitivity Questionnaire 3. Attitude towards shared decision-making scale	1. There was no significant difference between two groups in EOL care performance, although the increase in the interventional group was higher than that of the group. 2. Experimental group showed significantly higher scores in moral sensitivity and attitude towards shared decision-making after the intervention than the control group.
Lum, 2018 [22]	To develop and evaluate an ACP educational session on value-based ACP process	Pre-test post-test design	127 third-year medical students in the US	75-min value-based ACP educational session containing group discussion and watching an online video	Surveys on assessment of participants’ personal experiences with ACP conversations	1. 65% reported prior ACP conversations. After the intervention, 73% reported plans to discuss ACP, 91% had thought about preferences for future medical care, and 39% had chosen a medical decision maker. 14% completed an AD, and 1% talked with their health-care provider. 2. One month later, there was no evidence that the session increased students’ actions regarding these same ACP action steps.
Murray, 2010 [23]	To evaluate the effect of a program to train clinicians to support patients making decisions about place of EOL care	RCT	88 practitioners (majority nurses) from 7 community-based organizations and 3 hospital-based institutions in Canada	1. Intervention group: received six-week education program to target identified barriers to providing decision support for place of EOL care and improve decision support knowledge and skills by online tutorial, skill-building workshop and educational outreach 2. Control group: no education programme	1. Decisional Support Analysis Tool (DSAT10) 2. 10-item multiple choice questionnaires assessing knowledge of decisional support 3. Length of interaction in minutes 4. 31-item Factors Influencing Health Professionals Providing Support for Patients Preparing to Make Health Decisions survey tool	1. Compared to the control group, the intervention group had significantly greater improvement in DSAT10. 2. The intervention group scored significantly higher on the knowledge test than the control group. 3. The mean call duration was longer in the intervention group than the control group after the intervention. 4. The tool was rated as acceptable and clinically useful.
Seal, 2007 [24]	To evaluate the effect of the Respecting Patient Choices Program (RPCP) on fostering patient advocacy, promoting quality EOL assurance and associated job satisfaction	Prospective non-randomised control trial using convenience sampling and quasi-experimental and semi-structured focus group methods	278 nurses working in an acute care public hospital in South Australia	1. Intervention group: received RPCP focusing on quality of ACP process 2. Control group: no RPCP	1. 5-point Likert scales questionnaire 2. Focus group	1. There were statistically significant improvements in (1) encouragement to ensure patients could make informed choices about their EOL treatment, (2) the ability to uphold these wishes in practice, and (3) job satisfaction from delivering appropriate EOL care by nurse after RPCP program. 2. Focus group participants shared that it used to be hard to advocate for patients, but now they could act legitimately and felt ethically comfortable about ensuring EOL care.
Smith, 2013 [25]	To assess the feasibility and impact of a novel resident curriculum in EOL education to improve resident comfort with communication at EOL	Pre-test post-test design	165 internal medicine residents in the US	Two one-hour lunch conference sessions on EOL communication with didactic slides and scripted role play, and six one-hour morning reports focusing on discussion of real-time cases	Electronic survey with 24 questions related to demographics, previous requests for palliative care consultation, number of family meetings led, comfort with topics related to EOL care, behaviour during family meetings to discuss EOL care, and measures of self-efficacy for communication	The curriculum impacted resident reports of comfort with specific topics in EOL care, including discussions of code status and comfort care. Small impact on resident reports of self-efficacy for communication was also shown.
Wilson, 2017 [26]	To improve staff and family satisfaction with EOL communication and to increase the level of knowledge and the confidence of providers in discussing EOL issues in ICU through the quality improvement project	Pre-test post-test design	21 critical care staff (12 registered nurses, 4 acute care nurse practitioners, 1 social worker, 2 palliative care team members, 1 member from the spiritual care team, and 1 care manager) in the US	The first intervention was an educational program dealing with EOL communication, which was completed along with introduction of the standardised family meeting tool. The second was implementation of the new meeting format with documentation of the meeting outcomes in a standardised chart form.	1. Questionnaire was developed to assess healthcare providers’ knowledge, comfort level and experience of family meetings. 2. Chart review was conducted to document the variations and lack of consistency of family meetings.	1. Improved rating in perception of level of adequate training was found. There was an improvement in level of comfort after education. 2. Chart review demonstrated improvement in all areas of the team meeting documentation, indicating potential improved communication with the family and between care providers throughout all shifts.

*ACP* advance care planning, *AD* advance directives, *DPAHC* durable power of attorney for health care, *DSAT10* Decision Support Analysis Tool, *EOL* end-of-life, *ESKD* end-stage kidney disease, *GP* general practice, *ICU* intensive care unit, *RCT* randomized controlled trial, *RPCP* Respecting Patient Choices Program, *UK* United Kingdom, *US* United States

### Study interventions

All included studies involved instructional sessions in their interventions [17–26], while the interventions in seven studies contained discussions among participants [17–20, 22, 23, 25], and four included role-play [17–19, 25]. Advanced technology, such as audio-visual materials [17, 19, 20, 22], online tutorials [23] and interactive patient e-simulation [19], were also used. Regarding the training content, the majority focused on ACP communication skills [17, 21, 25, 26] and the needs and experience of patient in the ACP process [17, 18, 22]. Intensity of the intervention varied from 75-min values-based ACP educational session (Lum et al. [22]) to 2–3 h seminar /workshop (Greenberg et al. [20]), Detering et al. [19]), 8 h (Jo and An [21], Smith [25]), 2 days training (Alexander et al. [17], Bristowe et al. [18], Seal [24]) over a duration of 4 to 8 weeks (Jo and An [21], Murray et al. [23], Wilson et al. [26]). Of the 10 studies, only two studies (Alexander et al. [17] and Murray et al. [23]) scored high quality using EPHPP. As for program structure, Alexander et al. [17] used a short course (two-day retreat/ 16-h curriculum) to improve physicians’ communication skills in delivering bad news and eliciting patients’ preferences for EOL care. It has three components: control of pain and symptom management; communication skills; and sessions designed to promote participants’ understanding of patients and families’ experience, enhance their personal awareness, and inform them about ethical issues. Murray et al. [23] also used three components in the intervention – online tutorial 10 modules with quizzes and feedback; skill-building workshop with use of performance feedback, video exemplar, place of care patient decision aid, case studies, and practice and feedback; and educational outreach. The educational program of Jo and An [21] included a series of eight sessions of 60 min, twice a week for 4 weeks. Each session was designed with a specific topic related to shared decision-making and determined by an expert panel [21]. The study by Seal [24] was mainly targeted at nurses and the intervention group received the Respecting Patient Choices Program (RPCP), which was part of a national palliative care program. Lum et al. [22] applied ‘Conversation Starter Kits’, a free downloadable handout emphasizing a values-based rather than a procedure-based ACP process. Bristowe et al. [18] conducted a survey on end-stage kidney disease patients in order to identify the specific needs of those participants cared for before the program. The educational intervention by Smith et al. [25] consisted of two one-hour ‘lunch conferences’ involving role-play, and six one-hour morning sessions discussing real-time cases. The participants in the high-intervention group of Greenberg et al. [20] were required to initiate AD discussion with a patient, family member or friend to apply what they had learnt in the earlier seminar. To train healthcare professionals facilitating the ACP process, a novel feature of the intervention of Murray et al. [23] was that it involved a specific patient decision aid (PtDA) for practitioners to guide patients in place of EOL care discussions, although the PtDA only focused on the place of care decision making instead of a broader coverage of all relevant EOL healthcare decision-making in the ACP process. Wilson et al. [26] involved the use of standardized family meeting tool with documentation of the meeting outcomes in the intervention to improve EOL communication.

### Outcome measures

The outcome measures of the studies were mainly trainee-related outcomes, including knowledge, competence and skills in ACP after the educational program. In the study by Murray et al. [23], the primary outcome measure was the change in the quality of decision support provided by practitioners to a standardized patient before and after the intervention, while Alexander et al. [17] also used standardized patients to evaluate residents’ communication skills. The use of a standardized patient to collect evaluation data is more objective than a self-assessed performance, as self-reported changes are unverifiable. Secondary outcome measures used by Murray et al. [23] were knowledge, duration of interaction, intention to engage in patient decision support and acceptability of intervention components, including the decision tool, which were comprehensive and reflected the effectiveness of the training program from different aspects. A substantial number of studies adapted validated tools, e.g. Decision Support Analysis, Factors Influencing Health Professionals Providing Support for Patients Preparing to Make Health Decisions, Bed-News Conversations and Patient Preferences, to measure the outcomes. Jo and An [21] used the end-of-life care performance scale developed by Park [27], the Moral Sensitivity Questionnaire developed by Lutzen et al. [28], and attitudes towards shared decision-making scale developed by Jo as their outcome measures. A semi-structured focus group was used by Seal [24] to give qualitative data on participants’ perceptions of the program. The least productive of evidence was the use of non-validated questionnaires/surveys as in the studies of Bristowe et al. [18], Detering et al. [19], Greenberg et al. [20], Seal [24] and Lum et al. [22, 25]. Except Alexander et al. [17], Smith et al. [25], and Wilson et al. [26], all mentioned about the time course of their follow up measurements from immediately post training to 2 weeks (Detering et al. [19], Murray et al. [23]), 4 weeks (Jo and An [21], Lum et al. [22]), 6 weeks (Greenberg et al. [20]), 3 months (Bristowe et al. [18]), and 6 months (Seal [24]) after training. All except Lum et al. [22] reported intervention effect maintained at time of follow up. Lum et al. [22] reported no evidence that the training session increased participants’ actions regarding ACP action steps one-month post training. They commented that the one-month follow up period may not have provided adequate time for the participants to take ACP action, which is a limitation of such study design.

### Effects of interventions

All the included intervention studies showed positive results. The training programs significantly increased knowledge [20, 23], attitudes towards shared decision-making [21], perceived communication skills [17, 20, 23], confidence [19, 25], comfort [20, 25, 26] and experiences [20] in discussing EOL issues. Bristowe et al. [18] and Jo and An [21] reported non-significant increases in confidence in EOL communication and performance respectively. Wilson et al. [26] found improvement in all areas of team-meeting communication after intervention. The mean duration of interaction with standardized patients was longer in the intervention than the control group after the intervention by Murray et al. [23]. Lum et al. [22] found that 90% of participants evaluated the educational value of the session positively. However, there was no evidence that the session increased students’ actions regarding the ACP steps after 1 month. Lum et al. [22] explained that the one-month follow-up period might not provide the students with adequate time to take ACP action. The study by Seal [24] fostered patient advocacy by nurses and associated job satisfaction, as nurses thought that they could deliver appropriate EOL care to patients after the program. Improved rating of perceived levels of adequate training for EOL care was found by Wilson et al. [25, 26]. The study by Murray et al. [23] revealed that participants welcomed the use of decision tools in ACP communication, as they were rated as acceptable and clinically useful.

### Methodological quality

Detailed information on the methodological quality of each included study is presented in Table 2. Quality assessment of the ten studies using EPHPP showed two to be strong [17, 23], two moderate [21, 25] and six weak [18–20, 22, 24, 26] in the global rating. Concerning selection bias, one [24] was weak as less than 60% of the selected individuals agreed to participate. Of the other nine studies, six [17–22] had 80–100% agreement. As for study design, two were RCTs [20, 23], two were quasi-experimental with a non-equivalent control group pretest-posttest design [17, 21], one was a prospective non-randomized control trial using convenience sampling and quasi-experimental and semi-structured focus group methods [24], and the others were cohort (one group pre & post) [18, 19, 22, 25, 26]. Except the study conducted by Detering et al. [19], there were no important differences between groups prior to the intervention, or no control group was presented in all other studies. The outcome assessors were not blinded to the intervention or exposure status of participants in the studies of Greenberg et al. [20], Jo and An [21], Lum et al. [22], Seal [24], Smith et al. [25] and Wilson et al. [26]. Except for Bristowe et al. [18], Detering et al. [19], Greenberg et al. [20], Seal [24] and Lum et al. [22], where the validity of the data collection tools was not mentioned, all the other studies showed valid data collection tools. All except Seal [24] reported attrition numbers, and in that study the percentage of participants completing the program was less than 60%.

**Table 2:** Quality appraisal of included studies using the Effective Public Health Practice Project

Component	Description	First author, year																			
		Alexander, 2006 [17]		Bristowe, 2014 [18]		Detering, 2014 [19]		Greenberg, 1993 [20]		Jo, 2015 [21]		Lum, 2018 [22]		Murray, 2010 [23]		Seal, 2007 [24]		Smith, 2013 [25]		Wilson, 2017 [26]	
		A	R	A	R	A	R	A	R	A	R	A	R	A	R	A	R	A	R	A	R
A) Selection bias	Q1) Are the individuals selected to participate in the study likely to be representative of the target population? (1: Very likely; 2: Somewhat likely; 3: Not likely; 4: Can’t tell)	2	2 (M)	2	2 (M)	2	2 (M)	2	2 (M)	2	2 (M)	2	2 (M)	2	2 (M)	1	3 (W)	2	2 (M)	2	2 (M)
	Q2) What percentage of selected individuals agreed to participate? (1: 80–100% agreement; 2: 60–79% agreement; 3: Less than 60% agreement; 4: Not applicable; 5: Can’t tell)	1		1		1		1		1		1		2		3		2		5	
B) Study Design	Indicate the study design (1: RCT; 2: Controlled clinical trial; 3: Cohort analytic (two group pre + post); 4: Case-control; 5: Cohort (one group pre + post (before and after)); 6: Interrupted time series; 7: other specify; 8: Can’t tell)	2	1 (S)	5	2 (M)	5	2 (M)	1	1 (S)	3	2 (M)	6	2 (M)	1	1 (S)	3	2 (M)	5	2 (M)	5	2 (M)
	Was the study described as randomized? If NO, go to Component C.	N		N		N		Y		N		N		Y		N		N		N	
	If Yes, was the method of randomization described?							N						Y							
	If Yes, was the method appropriate?							N						Y							
C) Confounders	Q1) Were there important differences between groups prior to the intervention? (1: Yes; 2: No; 3: Can’t tell)	2	1 (S)	3	3 (W)	1	3 (W)	2	1 (S)	2	1 (S)	2	1 (S)	2	1 (S)	3	3 (W)	2	1 (S)	3	3 (W)
	Q2) If yes, indicate the percentage of relevant confounders that were controlled (either in the design (e.g. stratification, matching) or analysis)? (1:80–100% agreement; 2:60–79% agreement; 3: Less than 60% agreement; 4: Not applicable; 5: Can’t tell)	4		4		3		1		4		4		1		5		4		5	
D) Binding	Q1) Was (were) the outcome assessor (s) aware of the intervention or exposure status of participants? (1: Yes; 2: No, 3: Can’t tell)	2	2 (M)	3	3 (W)	3	3 (W)	1	3 (W)	1	3 (W)	1	3 (W)	2	2 (M)	1	3 (W)	1	3 (W)	1	3 (W)
	Q2) Were the study participants aware of the research question? (1: Yes; 2: No; 3: Can’t tell)	3		3		3		3		1		1		3		1		1		1	
E) Data Collection Method	Q1) Were data collection tools shown to be valid? (1: Yes; 2: No; 3: Can’t tell)	1	2 (M)	3	3 (W)	3	3 (W)	3	3 (W)	1	1 (S)	3	3 (W)	1	1 (S)	3	3 (W)	1	2 (M)	1	2 (M)
	Q2) Were data collection tools shown to be reliable? (1: Yes; 2: No; 3: Can’t tell)	3		3		3		3		1		3		1		3		3		3	
F) Withdrawals and Dropouts	Q1) Were withdrawals and drop-outs reported in terms of numbers and/or reasons per group? (1: Yes; 2: No; 3: Can’t tell; 4: Not applicable)	1	1 (S)	3	3 (W)	1	1 (S)	2	1 (S)	1	1 (S)	1	2 (M)	1	1 (S)	2	3 (W)	1	1 (S)	3	3 (W)
	Q2) Indicate the percentage of participants completing the study. (If the percentage differs by groups, record the lowest). (1:80–100%; 2:60–79%; 3: Less than 60%; 4: Can’t tell; 5: Not applicable)	1		4		1		1		1		2		1		3		1		1	
G) Intervention Integrity	Q1) What percentage of participants received the allocated intervention or exposure of interest? (1:80–100%; 2:60–79%; 3: Less than 60%; 4: Can’t tell)	1		1		1		1		1		1		1		3		1		1	
	Q2) Was the consistency of the intervention measured? (1: Yes; 2: No; 3: Can’t tell)	3		3		3		3		1		3		2		3		3		3	
	Q3) Is it likely that subjects received an unintended intervention (contamination or co-intervention) that may influence the results? (1: Yes; 2: No; 3: Can’t tell)	3		3		3		3		3		3		3		3		3		3	
H) Analysis	Q1) Indicate the unit of allocation (Community OR Organisation/institution OR Practice/office OR individual)	Ind		Ind		Ind		Ind		H		Ins		Ind		Wa		Ind		Ind	
	Q2) Indicate the unit of analysis (Community OR Organisation/institution OR Practice/office OR individual)	Ind		Ind		Ind		Ind		Ind		Ind		Ind		Ind		Ind		Ind	
	Q3) Are the statistical methods appropriate for the study design? (1: Yes; 2: No; 3: Can’t tell)	1		1		1		1		1		3		1		1		1		1	
	Q4) Is the analysis performed by intervention allocation status (i.e. intention to treat) rather than the actual intervention received? (1: Yes; 2: No; 3: Can’t tell)	2		1		1		2		2		2		2		2		2		3	
Global rating		1 (S)		3 (W)		3 (W)		3 (W)		2 (M)		3 (W)		1 (S)		3 (W)		2 (M)		3 (W)	

*A* answer, *C* community, *H* hospital, *Ind* individual, *Ins* institution, *M* moderate, *N* no, *R* rating, *S* strong, *W* weak, *Wa* ward, *Y* yes

## Discussion

### Implications for clinical practice

The studies included in this review showed that ACP education or training for healthcare professionals has positive effect on the knowledge, attitude, skills and comfort of participants in discussing issues related to EOL decision-making. With adequate training and skills transfer, doctors and nurses in non-palliative care settings can be equipped with the appropriate attitudes, knowledge and skills for conducting ACP, to address patients’ and their families’/carers’ needs and preferences regarding their care. However, not many studies measured the effect of ACP facilitator training programs on frequency of initiating ACP discussions and there was a lack of strong evidence for an effect of ACP facilitator training programs on frequency of initiating ACP discussions. Lund et al. [12] underscored the impact of clinical and organizational pressures on implementation of ACP. Multiple and competing demands of other work, problems in sharing decisions and preferences of patients within and between healthcare organizations, and the availability and preparation of staff are the barriers highlighted that affect opportunities and frequency to initiate and operationalize ACP discussions in clinical settings. Barnes [29], Ke et al. [30], Johnson, Butow, Kerridge and Tattersall [31] also pointed out that clinical doctors and nurses in acute settings have a heavy workload and insufficient time to conduct ACP, especially when they have other clinical tasks to undertake; and ACP is also influenced by the organizational culture. Future study should explore ways to tackle the barriers and more studies should measure the effect of ACP facilitator training programs on frequency of initiating ACP discussions. Appropriate use of decision aids may be explored, to be used in clinical settings to assist the ACP discussion. Simple decision-making tools may likely increase the likelihood of their adoption and normalization in practice as well as increase patients’ willingness to engage with them [12]. Organizational support is a key success factor in implementing an ACP facilitator training program for healthcare professionals working in non-palliative care hospital settings. Organizational support entails the organizational commitment to identifying, documenting, sharing and acting upon patients’ preferences [12], promulgation of ACP facilitation in all settings, encouraging the incorporation of ACP discussions into the clinical practice of frontline clinicians through clinical governance procedures, and providing training to clinicians on ACP facilitation. It is foreseen that extensive discussion with hospital administrators and managers will be necessary for an additional workforce to implement ACP and to release frontline clinicians to receive the training program. Regular in-service training and workshops on ACP should also be arranged for newly joined clinical staff to ensure the sustainability of ACP discussion as a normal practice in non-palliative acute care settings.

### Implications for future research

The results of this review also showed that there were limited high quality RCTs conducted internationally to evaluate the effectiveness of ACP facilitator training programs for healthcare professionals working in non-palliative care hospital settings. Although one of the included studies used decision tools to train practitioners facilitating ACP discussion [23], the systematic review by Cardona-Morrell et al. [14] implied that decision aids for initiating or terminating EOL treatment were scarce and lacked prognostic information on the pros and cons of alternative treatment options and preferences. More systematic evaluation of the effectiveness of a comprehensive decision aid is needed, aid that includes patient values (such as quality EOL, physical functioning, burden on families and attitudes to risk) and other important information like prognosis, treatment goals, and pros and cons of treatment, to guide patients and their surrogates to make EOL decisions, so that decision aids may be included to facilitate communication and understanding of the patient experience in future ACP facilitator training programs.

### Recommendations for the intervention to be developed

In summary, there is limited interventional study on training healthcare professionals from hospital settings as ACP facilitators, but there is a great demand for it [3, 22]. Training programs on ACP discussions should be considered that include teaching sessions containing small group discussion and communication skills training/workshops providing opportunities to practise the specific skills through role play with the use of appropriate decision aids [14, 17, 23]. Advanced technology such as interactive patient e-simulation can be used to provide more practice for trainees [19]. Values-based instead of procedure-based ACP educational sessions should be adopted. The content of the Conversation Starter Kit could be used as the frame for values-based ACP discussion [22], as it is a free downloadable handout, which makes it more accessible. The Respecting Patient Choices Program (RPCP) can be considered as a model for training nurses [24]. The systematic review by Myers et al. [32] found that Respecting Choices or tools based on that program were the ACP provider tools most often encountered in the literature, and suggested it was a critical strategy for affecting patient outcomes. Furthermore, similar to the study by Bristowe et al. [18], patient surveys can be conducted to identify the needs of patients that the target participants serve, and discussion with patient, family member or friend can be fostered to apply what the participants have learnt from the training program [20].

As for outcome measurement, qualitative data on patients and families’ perceptions of and satisfaction with ACP implementation by healthcare professionals by structured interview can be considered.

The relatively short, intensive course in Alexander et al. [17] yielded statistically significant positive results, and so may be taken as a reference for the intervention design of the future training program. However, Alexander et al. [17] designed the course mainly for US medical residents. The eight training sessions developed by Jo and An [21] for Korean nurses may also be considered as a reference. A qualitative interview is needed to understand healthcare professionals’ perceptions of ACP implementation by clinicians working in non-palliative care hospital settings to inform the development of the training program.

### Strengths and limitations

This is the first systematic review evaluating the effects of ACP facilitator training programs for healthcare professionals. The methodology adhered to the PRISMA statement [33] and the quality of each study was critically assessed using EPHPP [16].

Although each study contained clear descriptions of objectives, the intervention, outcome measurement and study finding, meta-analysis cannot be performed because the intervention and outcome measurements are so varied. However, positive results from all the studies indicated that ACP facilitator training programs for healthcare professionals were effective in increasing their knowledge, attitudes and skills in ACP.

## Conclusions

Communication and decision-making about the goals of care are identified by seriously ill hospitalized patients and their families as important targets for improvement, if the quality of EOL care is to be enhanced [2]. Although a significant proportion of deaths occur in non-palliative care settings, clinicians in acute settings have low involvement in ACP discussions [9]. The importance of ACP in clarifying patients’ values and respecting their wishes or autonomy is clear. Nurses have a valuable role in leading ACP implementation and in creating system-wide cultural changes to improve EOL care [34]. This systematic review found that training for healthcare professionals in ACP had positive effects on their knowledge, attitude and skills. However, there is a lack of high quality RCTs to evaluate the effectiveness of ACP facilitator training programs for nurses working in non-palliative care hospital settings. By evaluating the effectiveness of the training programs, the possibilities of such programs in clinical practice will be explored and recommendations for further development of ACP training program will be made, to enhance quality EOL care in non-palliative care hospital settings. In conclusion, the use of decision aids and advanced technology, instructional sessions with role play, training content focused on ACP communication skills and the needs and experience of patient in the ACP process, and a values-based ACP process are all those factors that made the ACP training programs effective.

## Data Availability

All data generated or analyzed during this study are included in this published article.

## Abbreviations

ACP: Advance care planning
AD: Advance directive
EOL: End-of-life
EPHPP: Effective public health practice project
PtDA: Patient decision aid
RCT: Randomized controlled trials
RPCP: The Respecting Patient Choices Program
